# Avoiding splenectomy in splenic sclerosing angiomatoid nodular transformation through endoscopic ultrasound-guided tissue acquisition: a 36-month follow-up case report

**DOI:** 10.1007/s12328-025-02252-6

**Published:** 2025-11-27

**Authors:** Takaki Okuyama, Kazuyuki Matsumoto, Kosaku Morimoto, Shogo Kimura, Takayoshi Miyake, Takuya Satomi, Kensuke Takei, Shogo Inoue, Ryuta Takenaka

**Affiliations:** 1 Department of Internal Medicine, Tsuyama Chuo Hospital, Kawasaki, Tsuyama City, 1756, 708-0841 Okayama Japan; 2 Department of Gastroenterology and Hepatology, Okayama University Hospital, 2-5-1 Shikata-cho, Kita-ku, Okayama City, 700-8558 Okayama Japan; 3 Department of Pathology, Tsuyama Chuo Hospital, Kawasaki, Tsuyama City, 1756, 708-0841 Okayama Japan

**Keywords:** Sclerosing angiomatoid nodular transformation, Spleen, Endoscopic ultrasound-guided tissue acquisition, Conservative management, Biopsy

## Abstract

A 48-mm splenic mass was incidentally discovered in a 78-year-old man upon computed tomography. Follow-up imaging at 12 months revealed enlargement to 60 mm, prompting endoscopic ultrasound-guided tissue acquisition with a 22-gauge needle. Histopathological analysis confirmed that it was a sclerosing angiomatoid nodular transformation. The patient was asymptomatic and had no hematologic abnormalities; therefore, splenectomy was not performed. After biopsy, the lesion regressed from 60 mm to 46 mm, possibly owing to hematoma formation or vascular disruption, and remained stable during 36 months of follow-up. Although splenectomy has been performed in most reported cases of sclerosing angiomatoid nodular transformation because of diagnostic uncertainty, a few recent reports have demonstrated that sclerosing angiomatoid nodular transformation can be diagnosed by endoscopic ultrasound-guided tissue acquisition, thereby avoiding splenectomy. This case highlights the diagnostic utility of endoscopic ultrasound-guided tissue acquisition and supports spleen-preserving management for biopsy-proven sclerosing angiomatoid nodular transformation.

## Introduction

Sclerosing angiomatoid nodular transformation (SANT) is a rare, benign vascular lesion of the spleen, first described by Martel et al. [1]. Most cases are asymptomatic and detected incidentally, although some patients report nonspecific abdominal discomfort. Hematologic abnormalities—such as anemia or thrombocytopenia—are uncommon and may be resolved by splenectomy [2, 3].

Histologically, SANT is well-demarcated and multinodular, with angiomatoid nodules in fibrosclerotic stroma. Immunohistochemistry reveals a characteristic triphasic vascular immunophenotype—capillary-like, sinusoid-like, and small-vein-like—indicative of a reactive, non-neoplastic process [4]. This signature facilitates its distinction from other splenic vascular tumors. Although several imaging patterns have been described, imaging is not pathognomonic. Upon contrast-enhanced computed tomography (CT), SANT typically exhibits peripheral-predominant enhancement with progressive centripetal enhancement over multiple phases. Upon magnetic resonance imaging (MRI), a “spoke-wheel” pattern with a central scar may be observed. However, these features are variable and non-specific, and they do not reliably distinguish SANT from malignant or other benign vascular splenic lesions; therefore, histopathologic confirmation is usually required [5–7].

Many patients undergo splenectomy for a definitive diagnosis; however, splenectomy entails non-negligible risks. Minimally invasive tissue acquisition—particularly endoscopic ultrasound-guided tissue acquisition (EUS-TA)—is a safe and accurate alternative that enables spleen-preserving management in appropriate candidates, although only one report of EUS-TA has been published [8]. Herein, we report a case of splenic SANT that was diagnosed via EUS-TA; the patient was managed conservatively, and a transient post-biopsy reduction in size was observed. Asymptomatic patients with pathologically confirmed SANT may avoid splenectomy. Our findings suggest that splenectomy may be avoided in asymptomatic patients with pathologically confirmed SANT.

## Case report

A 78-year-old man underwent abdominal CT after a fall, which incidentally revealed a well-circumscribed, ovoid splenic mass that measured 48 mm in diameter. He was asymptomatic at the time of detection. Laboratory tests revealed a hemoglobin (Hb) concentration of 14.4 g/dL, white blood cell (WBC) count of 8600/µL, and platelet count of 179,000/µL. The patient’s C-reactive protein (CRP) concentration was mildly elevated, at 0.84 mg/dL. His liver function parameters—including aspartate aminotransferase (31 U/L), alanine aminotransferase (35 U/L), alkaline phosphatase (55 U/L), and total bilirubin (1.4 mg/dL) concentrations—were within or near the normal ranges. His tumor markers were also within the reference ranges: carcinoembryonic antigen concentration, 2.4 ng/mL (< 5.0); squamous cell carcinoma antigen concentration, 2.6 ng/mL (< 1.5); and CYFRA 21 − 1 concentration, 3.2 ng/mL (< 3.5). Coagulation test results were normal (prothrombin time–international normalized ratio: 0.99; activated partial thromboplastin time: 24.2 s).

**Fig. 1:**
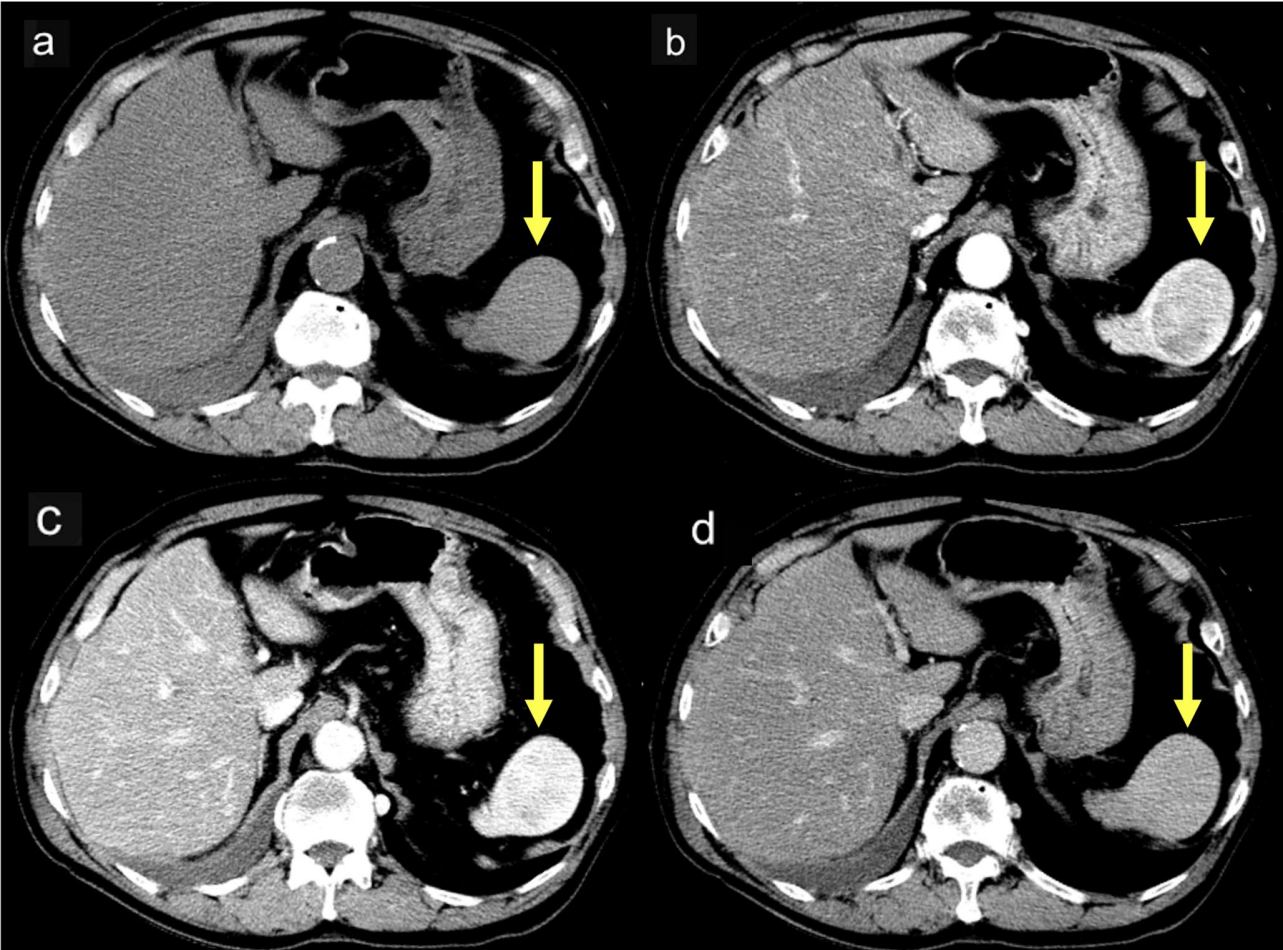
**a** Non-contrast CT reveals a well-defined splenic mass, isoattenuating to the surrounding splenic parenchyma. **b** Arterial phase of contrast-enhanced CT demonstrates peripheral-predominant, heterogeneous enhancement. **c** Portal venous phase of contrast-enhanced CT reveals progressive centripetal enhancement with mild residual heterogeneity. **d** Delayed phase of contrast-enhanced CT demonstrates near-homogeneous attenuation approximating that of the surrounding parenchyma. The lesion is indicated by yellow arrows. CT: computed tomography

**Fig. 2:**
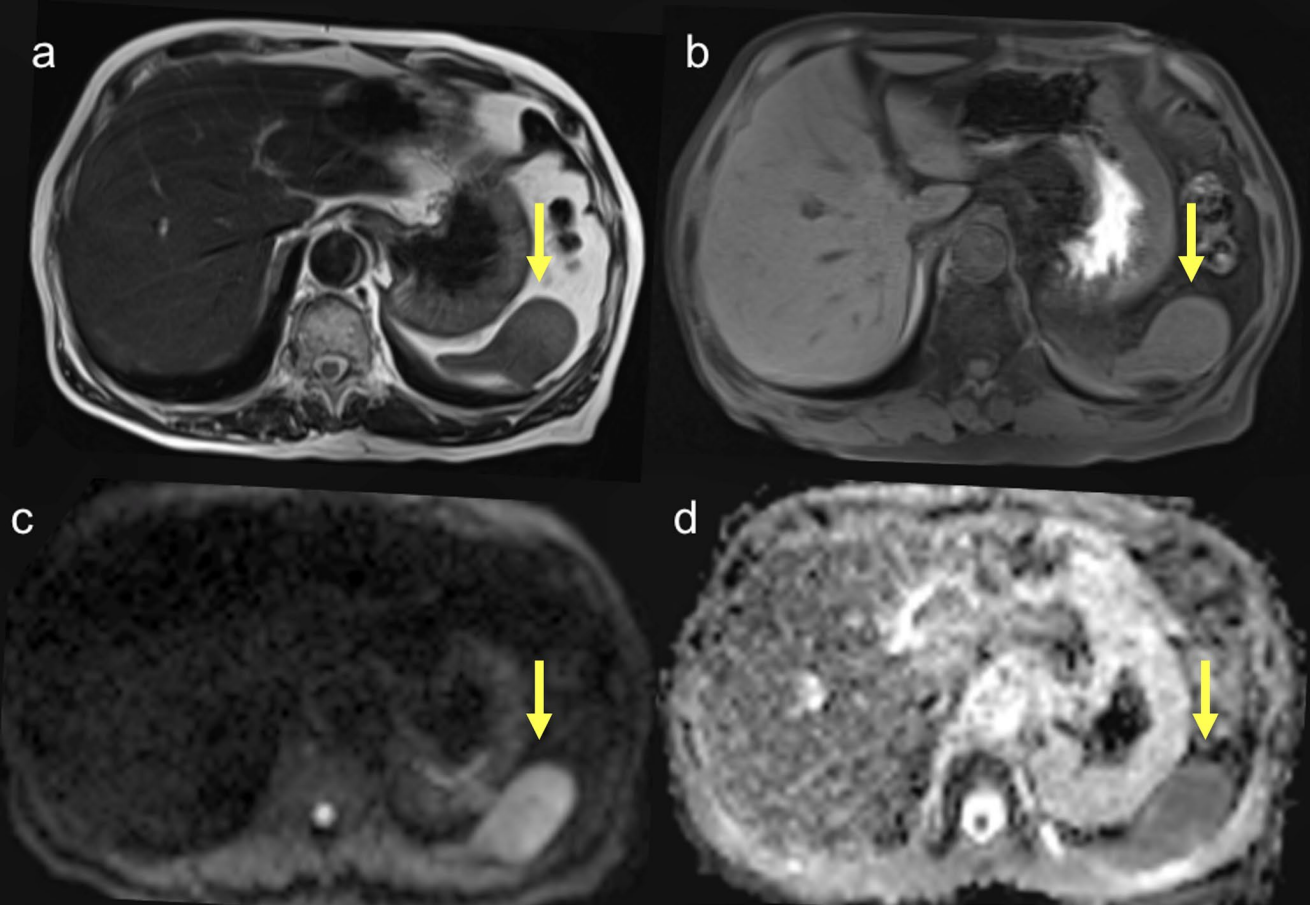
**a, b** T1- and T2-weighted images reveal a well-defined splenic mass, predominantly isointense to the splenic parenchyma, with linear and patchy hypointense foci. **c** DWI (b = 1000 s/mm^2^) reveals a high signal. **d** ADC map reveals a low value, consistent with restricted diffusion. DWI: diffusion-weighted imaging; ADC: apparent diffusion coefficient. The lesion is indicated by yellow arrows

**Fig. 3:**
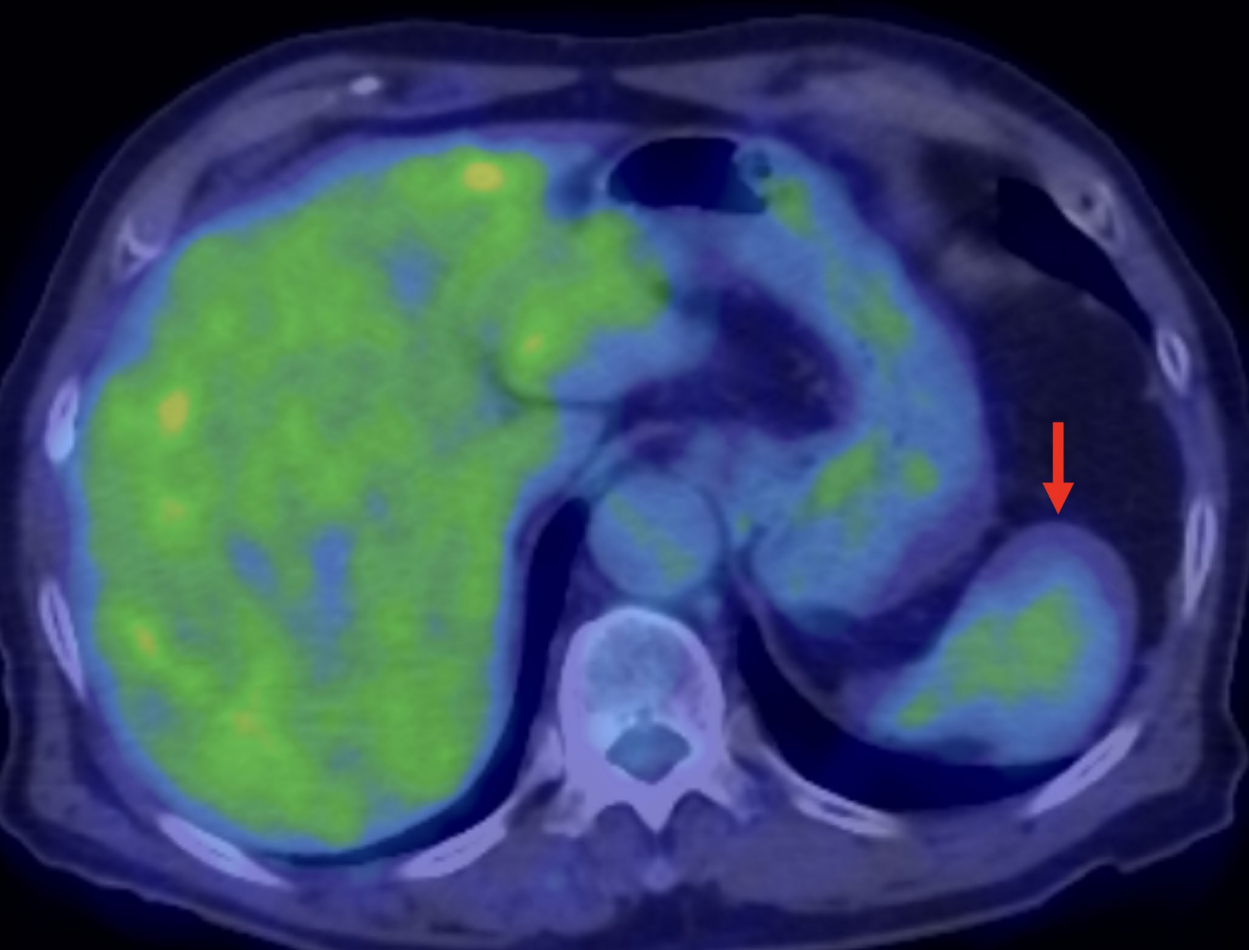
The splenic lesion demonstrates low-grade FDG uptake with a SUVmax of 2.32. FDG: fluorodeoxyglucose; SUVmax: maximum standardized uptake value. The lesion is indicated by red arrows

Upon non-contrast CT, the lesion appeared isoattenuating to the surrounding splenic parenchyma. Upon contrast-enhanced CT, it appeared as a well-defined splenic mass with progressive enhancement across the dynamic phases. The lesion exhibited peripheral-predominant and heterogeneous enhancement in the arterial phase, followed by centripetal progression of enhancement during the portal venous and delayed phases, ultimately resulting in relatively homogeneous attenuation (Fig. 1). The splenic capsule remained intact, with no evidence of perisplenic fat stranding, fluid collection, vascular invasion, or regional lymphadenopathy. Upon MRI, the lesion appeared isointense to the spleen on both T1- and T2-weighted sequences and demonstrated hyperintensity on diffusion-weighted images, with substantial restriction on the apparent diffusion coefficient map (Fig. 2). 18 F-fluorodeoxyglucose (18 F-FDG) positron emission tomography (PET)/CT revealed only mild FDG uptake, with a maximum standardized uptake value of 2.32 (Fig. 3), suggestive of low metabolic activity. Based on the absence of symptoms and imaging features suggestive of benignity, the differential diagnosis included vascular lesions such as a cavernous hemangioma, splenic hamartoma, SANT, and inflammatory pseudotumor. Metastatic disease (e.g., from lung carcinoma) was considered unlikely.

**Fig. 4:**
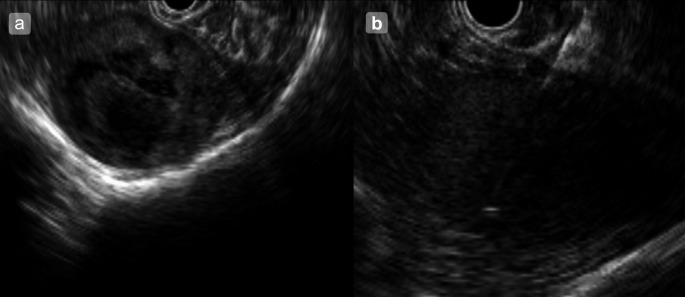
**a** EUS reveals a well-demarcated, hypoechoic splenic lesion with a heterogeneous internal echotexture. **b** EUS-TA was conducted using a 22-gauge Franseen-tip needle (Acquire; Boston Scientific, Marlborough, MA, USA); for the first pass, we used suction, but owing to initial blood contamination, subsequent passes were performed with the slow-pull technique; no immediate complications occurred. EUS: endoscopic ultrasound; EUS-TA: endoscopic ultrasound-guided tissue acquisition

Given the patient’s stable clinical condition and the absence of concerning clinical or hematologic findings, a conservative, observational approach was initially adopted. However, follow-up CT at 12 months revealed that the splenic mass had grown to 60 mm in diameter. In light of this progression and the need to exclude malignant entities such as lymphoma, angiosarcoma, and epithelioid hemangioendothelioma, EUS-TA (Fig. 4) was performed using a 22-gauge Franseen-tip needle (Acquire; Boston Scientific, Marlborough, MA, USA). The first pass was obtained with suction; owing to initial blood contamination, two additional passes were performed using the slow-pull technique to improve sample quality. The procedure and hospital course were uneventful. Vital signs remained stable, and laboratory values on post-procedure day 1 (Hb, WBC, CRP) were within normal limits. At the 3-month follow-up, the patient remained asymptomatic, with no anemia, no increase in inflammatory markers, and no other complications.

**Fig. 5:**
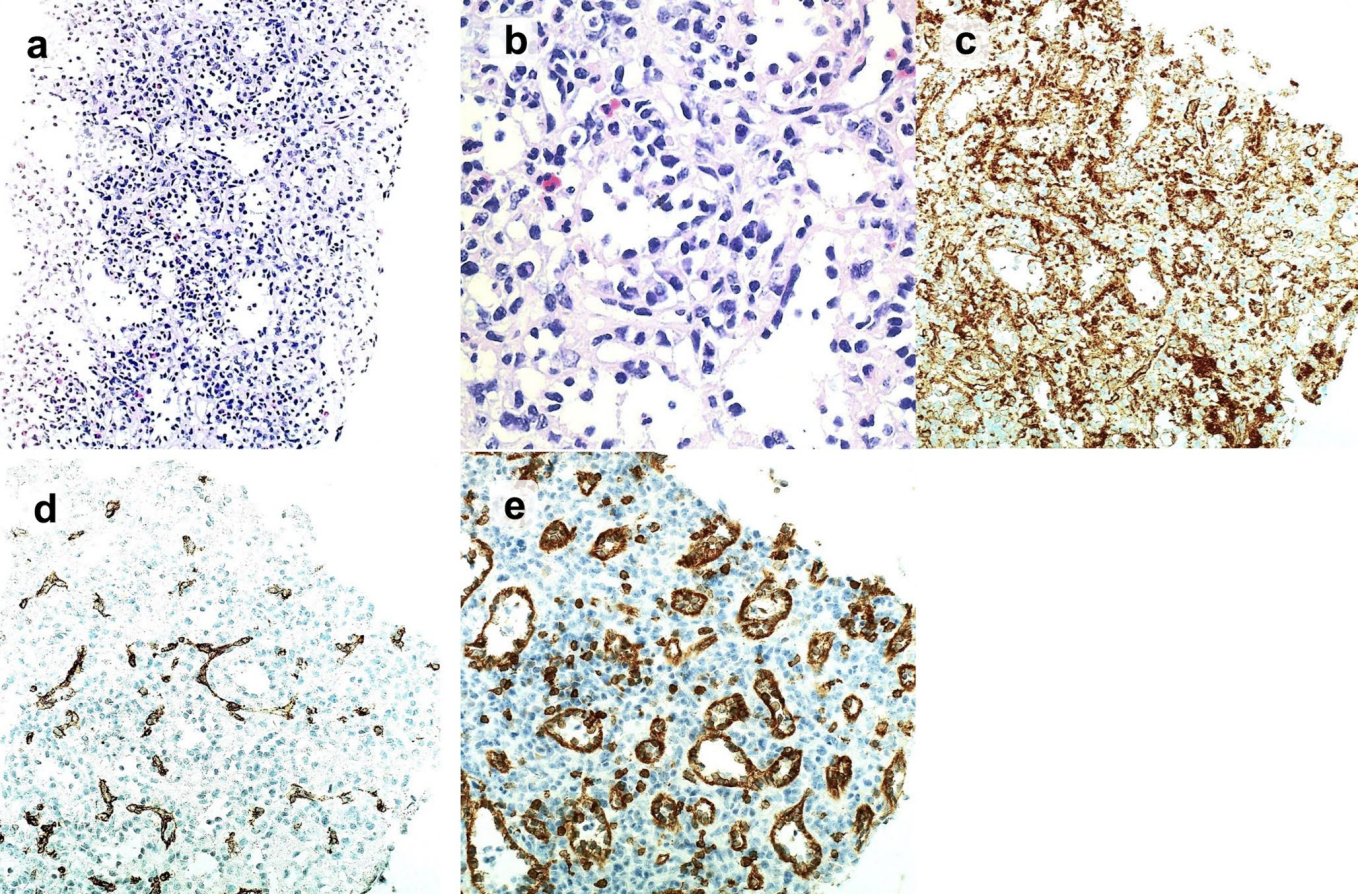
(**a** H.E. staining (low-power view) reveals multinodular angiomatoid proliferation within fibrosclerotic stroma, with slit-like vascular spaces (original magnification: ×100). **b** H.E. staining (high-power view) confirms a flattened, continuous endothelial lining without cytologic atypia or multilayering (original magnification: ×400). **c** CD31 immunohistochemistry yields diffuse membranous staining of endothelial cells (original magnification: ×200). **d** CD34 immunohistochemistry stains capillary-like vessels (original magnification: ×200). **e** CD8 immunohistochemistry stains sinusoid-like vessels (original magnification: ×200). H.E.: hematoxylin and eosin; CD: cluster of differentiation

Histopathological examination revealed multiple well-circumscribed nodules, separated by fibrosclerotic stroma; hematoxylin and eosin staining revealed prominent vascular spaces and intervening fibrosis within the nodules (Fig. 5a, b). Immunohistochemistry revealed the triphasic vascular immunophenotype characteristic of SANT: endothelial cells revealed diffuse, membranous staining with cluster of differentiation (CD)31 (Fig. 5c); capillary-like vessels were CD34-positive (Fig. 5d)/CD8-negative; sinusoid-like vessels were CD8-positive (Fig. 5e)/CD34-negative; and small vein-like vessels were CD31-positive only (Fig. 5c). These immunophenotypic findings, together with the characteristic histomorphology, supported the diagnosis of SANT.

**Fig. 6:**
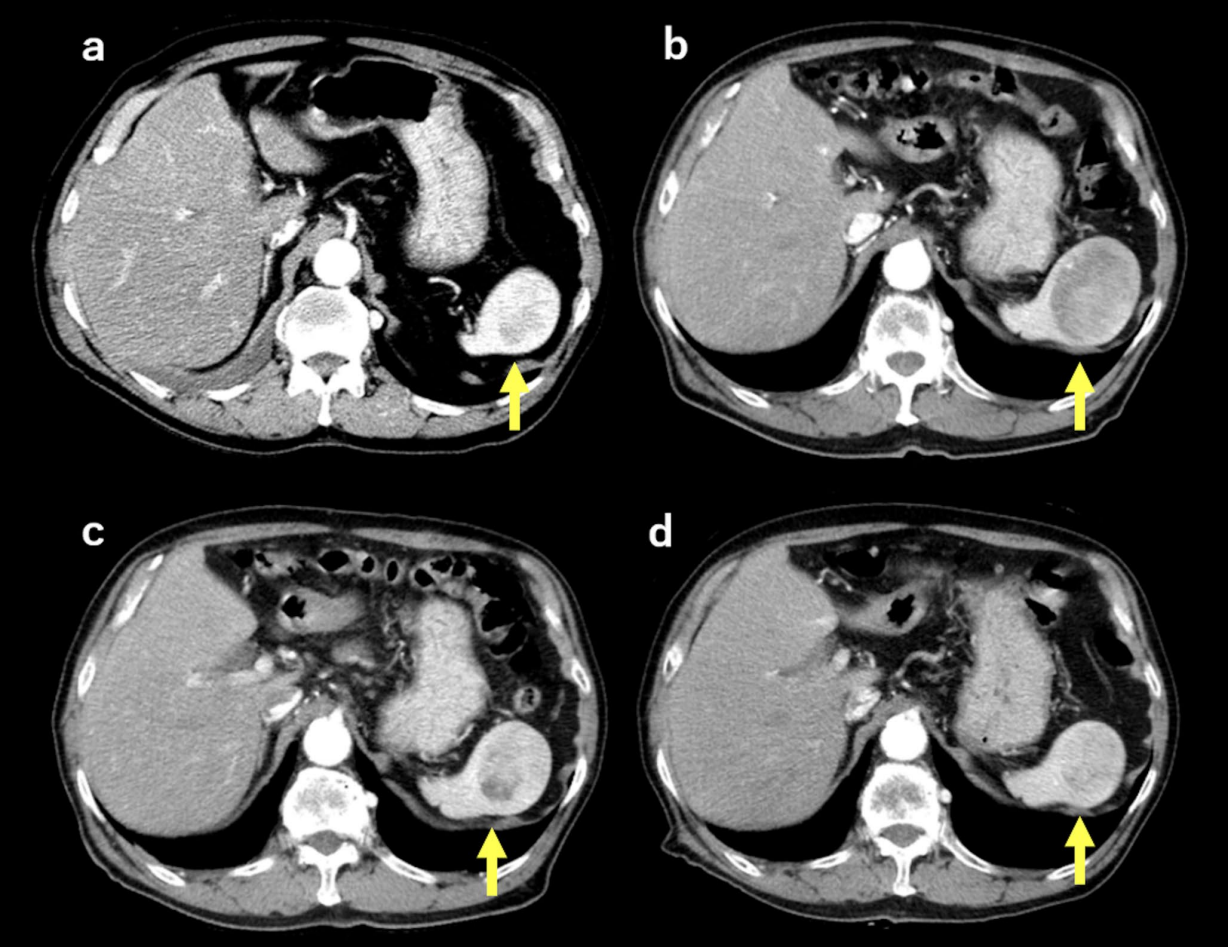
**a** Initial scan showing a 48 mm mass with mild heterogeneous enhancement. **b** Enlargement to 60 mm by the 12-month follow-up. **c** Three months after EUS-TA, the lesion decreased to 46 mm with newly developed, patchy hypoattenuation; these areas showed a mean attenuation of 54 HU, compatible with post-hemorrhagic change. **d** Stable lesion morphology at 36 months after EUS-TA. HU: Hounsfield units. The lesion is indicated by yellow arrows

Because of concerns about splenectomy-related complications, conservative management was adopted. Three months after EUS-TA, contrast-enhanced CT demonstrated interval shrinkage of the lesion to 46 mm, accompanied by newly developed, patchy hypoattenuation; these foci had a mean CT attenuation of 54 Hounsfield units (HU), compatible with post-hemorrhagic change. Thereafter, the lesion remained stable in size and configuration during the subsequent 36-month follow-up, with gradual resolution of the hypoattenuating areas (Fig. 6). The patient remained asymptomatic during the entire observation period.

## Discussion

This case highlights the clinical value of EUS-TA in establishing a definitive diagnosis of SANT for an enlarging splenic tumor that was difficult to differentiate from malignancy, thereby avoiding unnecessary splenectomy. Uniquely, follow-up imaging revealed tumor shrinkage, a phenomenon not previously reported in SANT, which may have been related to biopsy-induced intratumoral hemorrhage.

Although a benign entity such as SANT may be radiologically suspected, lesion enlargement often triggers splenectomy out of concern for malignancy [4, 9, 10]. However, interval growth has been documented in histologically confirmed SANT, with no documented instances of malignant transformation [11, 12]. Consequently, clinicians in most published cases have therefore proceeded to perform splenectomy largely owing to diagnostic uncertainty, despite its intraoperative and long-term risks, including hemorrhage, pancreatic injury, and overwhelming post-splenectomy infection [13]. In this context, recent reports increasingly support nonsurgical management in carefully selected patients—those who are asymptomatic, exhibit no hematologic abnormalities, and have histologically confirmed SANT [9, 11, 14]. EUS-TA is gaining recognition as a safe and reliable diagnostic modality for splenic lesions [15]. In hypervascular tumors such as SANT, the slow-pull technique can help to reduce blood contamination and improve sample quality [16]. In our case, this approach enabled histological confirmation without requiring splenectomy.

The adverse-event rate of EUS-TA for lesions of the gastrointestinal tract and adjacent organs is 0.98%, with a procedure-related mortality of 0.02% (51 studies, 10,941 patients) [17]. By contrast, for splenic lesions, an adverse-event rate of 4.7% has been reported (6 studies, 62 patients), with no procedure-related deaths [18]. This rate is relatively higher. Accordingly, the indication for splenic EUS-TA should be carefully evaluated. Because splenic EUS-TA carries inherent procedural risks, it should be reserved for cases in which a definitive diagnosis is necessary—specifically, for splenic lesions that lack characteristic imaging features (such as the spoke-wheel pattern) or demonstrate FDG uptake, where exclusion of other malignancies is essential. Furthermore, splenectomy carries substantial drawbacks, including increased risks of infection and thrombosis, with postoperative morbidity rates of 8.6%–37% and mortality rates of up to 2.9%; therefore, it should be avoided whenever possible. In the present case, EUS-TA enabled a definitive diagnosis of SANT, allowing conservative management without splenectomy.

To place our findings in context, we reviewed three previously reported cases of biopsy-proven SANT that were managed conservatively (without splenectomy), together with the present case (Table 1) [8, 19, 20]. The literature search was performed in PubMed (2004–2025) using the keywords “sclerosing angiomatoid nodular transformation,” “spleen,” and “biopsy.” In all four cases, patients were asymptomatic, exhibited no hematologic abnormalities, and histological confirmation was successfully obtained via either EUS-TA or percutaneous biopsy without any complications. Radiologically, most lesions were well-circumscribed, and in the three cases assessed via FDG-PET/CT, only mild uptake was observed. Moreover, the spoke-wheel pattern, although regarded as characteristic of SANT, was observed in only one case. These observations underscore the difficulty of imaging-based diagnosis, as emphasized previously [8, 12], showcasing the indispensable role of histological confirmation. However, the previous cases demonstrated radiologic stability during follow-up, whereas the present case exhibited post-biopsy lesion regression—a phenomenon not previously documented in SANT. This rare occurrence may reflect vascular disruption, hematoma formation, or other biopsy-induced changes, as reported for hypervascular tumors such as hepatocellular and renal cell carcinoma [21, 22]. Importantly, this observation suggests that biopsy can transiently alter lesion morphology. Post-biopsy regression has not been previously documented in SANT. This finding suggests that biopsy itself may alter tumor morphology and emphasizes the importance of careful follow-up imaging in biopsy-proven SANT that is managed conservatively.

**Table 1:** Reported cases of biopsy-proven Splenic SANT managed without splenectomy (2016–2025)

Authors (year)	Age	Sex	Symptoms	Spoke-wheel pattern^a^	PET/CT^b^	Lesion size	Diagnosis (needle gauge)	Complications	Follow-up
Dutta et al. (2016)	60	F	None	Present	SUVmax: 3.8	38 mm	US-guided Bx (not reported)	None	NA
Katsuda et al. (2021)	72	M	None	Absent	NA	34 mm	EUS-TA (FNA, 22G)	None	NA
Gómez-Rubio et al. (2025)	69	M	None	NA	SUVmax: 2.62	30 mm	US-guided Bx (18G)	None	10 years
Present case (2025)	78	M	None	Absent	SUVmax: 2.32	60 mm	EUS-TA (FNB 22G)	None	3 years

SANT, sclerosing angiomatoid nodular transformation; US: ultrasound, PET/CT: positron emission tomography/computed tomography, Bx: biopsy, FNA, fine-needle aspiration, EUS-TA: endoscopic ultrasound-guided tissue acquisition, NA: not available

^a^Spoke-wheel pattern: radiating fibrous septa or central scar upon CT or magnetic resonance imaging

^b^Upon PET/CT, mild fluorodeoxyglucose uptake is typically observed; the reported maximum standardized uptake values for SANT are generally 2–4, which are lower than those in malignant splenic tumors such as lymphoma or angiosarcoma [7]

This report also has several limitations to note. First, as it is a single case report, the observed post-biopsy regression may not be a common phenomenon. Second, the mechanism of regression remains speculative; our interpretation of hematoma formation or vascular disruption was based on imaging findings and on reports from biopsies of other hypervascular lesions but was not pathologically confirmed. Third, although the lesion remained stable over 36 months, its longer-term natural history remains unclear, and the possibilities of regrowth or late complications cannot be entirely excluded.

In conclusion, EUS-TA can provide a definitive diagnosis of SANT, thereby avoiding unnecessary splenectomy. Our case suggests that biopsy itself may influence tumor morphology.
